# Shaping leaves through TALE homeodomain transcription factors

**DOI:** 10.1093/jxb/erae118

**Published:** 2024-03-25

**Authors:** Mary E Byrne, Eleanor Imlay, Nazuratul Nabilah Binti Ridza

**Affiliations:** School of Life and Environmental Sciences, The University of Sydney, NSW 2006, Australia; School of Life and Environmental Sciences, The University of Sydney, NSW 2006, Australia; School of Life and Environmental Sciences, The University of Sydney, NSW 2006, Australia; University of Illinois, USA

**Keywords:** *Arabidopsis thaliana*, BLH, *Cardamine hirsuta*, compound leaf, KNOX, leaf shape, simple leaf, TALE homeodomain transcription factor, tomato

## Abstract

The first TALE homeodomain transcription factor gene to be described in plants was maize *knotted1* (*kn1*). Dominant mutations in *kn1* disrupt leaf development, with abnormal knots of tissue forming in the leaf blade. *kn1* was found to be expressed in the shoot meristem but not in a peripheral region that gives rise to leaves. Furthermore, KN1 and closely related proteins were excluded from initiating and developing leaves. These findings were a prelude to a large body of work wherein TALE homeodomain proteins have been identified as vital regulators of meristem homeostasis and organ development in plants. KN1 homologues are widely represented across land plant taxa. Thus, studying the regulation and mechanistic action of this gene class has allowed investigations into the evolution of diverse plant morphologies. This review will focus on the function of TALE homeodomain transcription factors in leaf development in eudicots. Here, we discuss how TALE homeodomain proteins contribute to a spectrum of leaf forms, from the simple leaves of *Arabidopsis thaliana* to the compound leaves of *Cardamine hirsuta* and species beyond the *Brassicaceae*.

## Teaser

TALE homeodomain transcription factors are important regulators of development. They have fundamental roles in determining leaf shape and are important in generating the rich diversity of leaf shape found in nature.

## Introduction

### Plant TALE homeodomain transcription factors

**Fig. 1.**
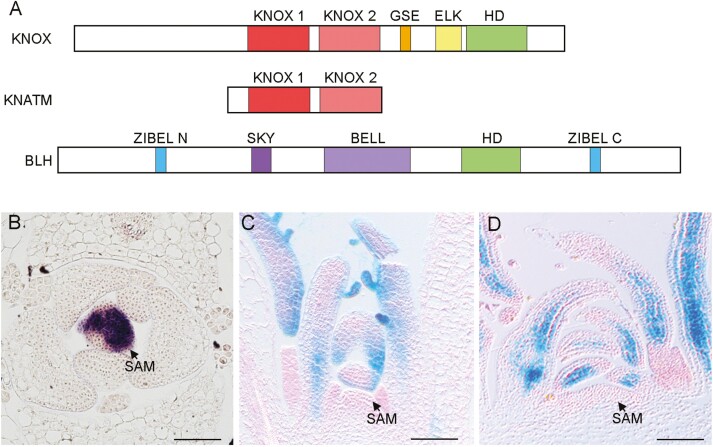
TALE homeodomain transcription factors in plants. (A) Schematic diagram of key domains in KNOX, KNATM, and BLH proteins. (B) The KNOXI gene *STM* is expressed in the shoot apical meristem (SAM) and is not expressed in initiating leaf primordia and leaves. (C) The KNOXII gene *KNAT3* (C) and the BLH gene *SAW1* (D) are expressed in the leaves and not in the shoot meristem. Scale bars indicate 50 µm.

Homeobox genes encode a 60 amino acid DNA-binding homeodomain with a characteristic protein fold composed of three α-helices connected by two short amino acid loops. Homeodomain proteins activate or repress transcription. The TALE (Three Amino Acid Loop Extension) homeodomain superclass is distinguished by three extra amino acids between helix 1 and helix 2 of the homeodomain (Bertolino *et al.*, 1995). KNOTTED1 (KN1) in maize is the founding member of TALE homeodomain transcription factors in plants (Vollbrecht *et al.*, 1991). TALE proteins in plants are encoded by KNOX (*knotted1*-like homeobox) and BLH (*BEL1*-like homeobox) genes, and are distinguished by the homeodomain sequence and the presence of conserved domains N-terminal to the homeodomain (Fig. 1). KNOX genes are further divided into two classes, class I KNOX (KNOXI) and class 2 KNOX (KNOXII) (Kerstetter *et al.*, 1994). KNOXI proteins have a diagnostic MEINOX domain, which is composed of two conserved domains, KNOX1 and KNOX2, separated by a variable region, as well as shorter GSE and ELK motifs adjacent to the homeodomain (Fig. 1A) (Vollbrecht *et al.*, 1991; Bharathan *et al.*, 1997; Bürglin, 1997). In many plants, KNOXI and KNOXII comprise a relatively small number of genes; for instance, *Arabidopsis thaliana* has four KNOXI and four KNOXII genes, and *Zea mays* has five KNOXI and eight KNOXII genes (Gao *et al.*, 2015). Genetic analysis indicates a degree of redundancy between members within a class, and phenotypes resulting from ectopic expression indicate that proteins within KNOXI and within KNOXII have somewhat different activities (Chuck *et al.*, 1996; Parnis *et al.*, 1997; Nishimura *et al.*, 2000; Byrne *et al.*, 2002; Furumizu *et al.*, 2015; Rast-Somssich *et al.*, 2015). The BLH class of proteins are uniquely characterized by two conserved domains upstream of the homeodomain, SKY and BELL, together called the MEINOX-interacting domain (MID). In addition, some BLH proteins have short conserved amino acid sequences, called ZIBEL, at both the N- and C-terminal ends of the protein (Fig. 1A) (Bellaoui *et al.*, 2001; Müller *et al.*, 2001; Becker *et al.*, 2002; Mukherjee *et al.*, 2009). The ZIBEL domain shares similarities with the ethylene-responsive element binding factor-associated amphiphilic repression (EAR) transcriptional repressor domain. A third TALE-related class of genes, class M KNOX, includes *KNATM* in *A*. *thaliana*. These genes encode proteins that have a MEINOX domain but lack the homeodomain (Fig. 1A) (Magnani and Hake, 2008).

KNOX and BLH proteins form protein–protein dimers mediated through the MEINOX and MID domains, respectively, although other domains potentially contribute to protein pairing. Heterodimerization can serve to increase affinity for binding target sequences, mediate nuclear localization, and increase specificity of the binding target sequence (Bellaoui *et al.*, 2001; Nagasaki *et al.*, 2001; Smith *et al.*, 2002; Chen *et al.*, 2003; Bhatt *et al.*, 2004; Cole *et al.*, 2006; Rutjens *et al.*, 2009; Liu *et al.*, 2014).  There is some degree of specificity of KNOX and BLH homodimer and heterodimer protein interactions, although the biological significance of such protein pairing, with some exceptions, remains largely undetermined (Müller *et al.*, 2001; Smith *et al.*, 2002; Byrne *et al.*, 2003; Smith and Hake, 2003; Hackbusch *et al.*, 2005; Furumizu *et al.*, 2015; Ezura *et al.*, 2022). KNOX and BLH protein interactions may be further influenced through protein pairing with KNATM (Kimura *et al.*, 2008; Magnani and Hake, 2008).

TALE homeobox genes are found in plants, animals, and fungi. This deep evolutionary history suggests that these genes were present in the last common ancestor of eukaryotes (Bharathan *et al.*, 1997; Bürglin, 1997, 1998). Heterodimer pairing between TALE homeodomain proteins in the unicellular green algae *Chlamydomonas* and the unrelated brown algae *Ectocarpus* mediates a haploid to diploid life cycle transition (Lee *et al.*, 2008; Arun *et al.*, 2019). In fact, KNOX and BLH proteins, which are deeply rooted in the *Viridiplantae*, are both required for development of the diploid sporophyte in the bryophytes *Marchantia polymorpha* and *Physcomitrella patens*. As such, it has been proposed that an ancestral function of TALE homeodomain transcription factors was to promote a haploid to diploid transition in plants (Sakakibara *et al.*, 2008, 2013; Mukherjee *et al.*, 2009; Hisanaga *et al.*, 2021). Furthermore, expansion of the TALE class of genes throughout the evolution of plants potentially increased the number of possible protein interactions available to regulate development and thereby promote more complex sporophyte body plans (Bowman *et al.*, 2016).

## Serrations in the simple leaves

**Fig. 2.**
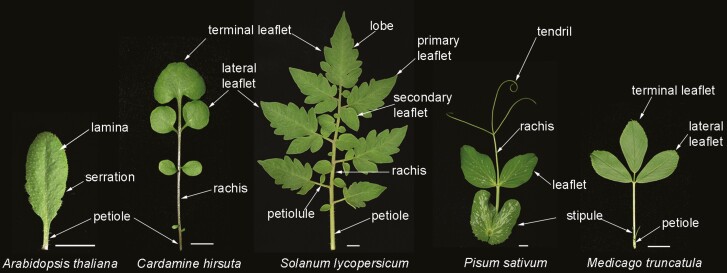
Simple and compound leaves. Different shapes of leaves with the basic anatomy labelled. From left to right: *Arabidopsis thaliana* simple leaf with serrations, *Cardamine hirsuta* pinnately compound leaf, *Solanum lycopersicum* bipinnately compound leaf, *Pisum sativum* pinnately compound leaf with terminal tendrils, and *Medicago truncatula* trifoliate compound leaf. Scale bars indicate 1 cm.

Angiosperm leaves are found in an enormous variety of shapes, but can fundamentally be considered as either simple, with a single continuous lamina, or compound, where the lamina is partitioned into separate leaflets (Fig. 2). The margin of leaf lamina may be further sculpted by differential growth, leading, for instance, to serrations or lobes. Leaves are typically flat structures with features for light interception and gas exchange to maximize photosynthesis. They emerge from the flanks of the shoot apical meristem and early in initiation define adaxial–abaxial (top–bottom), proximal–distal (base–tip), and medial–lateral (midvein to margin) axes that lead to growth of a flat organ. Cell growth and expansion build on this basic plan and modify shape through growth of secondary structures from meristematic regions, termed marginal blastozones, at the leaf margin (Hagemann and Gleissberg, 1996).

What we know about KNOX genes and how they affect leaf shape can be approached through an understanding of genetic regulatory mechanisms shaping simple leaves of *A. thaliana*. There are four well-studied KNOXI genes in *A. thaliana*, *SHOOT MERISTEMLESS* (*STM*), *BREVIPEDICELLUS/KNAT1* (*BP*), *KNAT2*, and *KNAT6*. All four genes are expressed in regions of the shoot meristem and, to varying degrees, contribute to maintaining the shoot meristem (reviewed in Maksimova *et al.*, 2021). *STM* is the most crucial of these genes, and loss-of-function mutations result in loss of the shoot meristem. *BP* and *KNAT2* are not essential for meristem function but act redundantly with *STM* (Byrne *et al.*, 2002; Belles-Boix *et al.*, 2006). All four KNOXI genes are down-regulated in cells of the initiating leaf primordium and, importantly, are not expressed in the developing leaf (Fig. 1B). This same KNOXI expression pattern, being present in the shoot meristem and down-regulated in initiating and growing leaves, is a common property of many angiosperm species that have simple leaves (Smith *et al.*, 1992; Lincoln *et al.*, 1994; Long *et al.*, 1996; Bharathan *et al.*, 2002; Groot *et al.*, 2005). KNOXI down-regulation in the initiating leaf primordium is at least in part mediated by the hormone auxin (Heisler *et al.*, 2005; Hay *et al.*, 2006; Heisler and Byrne, 2020). KNOXI are also repressed in developing *A. thaliana* leaves by multiple transcription and chromatin-remodelling factors (Satterlee and Scanlon, 2019; Jia *et al.*, 2023). Key transcription factors that repress KNOXI in the leaf are the MYB domain protein ASYMMETRIC LEAVES1 (AS1) and the LOB domain protein ASYMMETRIC LEAVES2 (AS2). Together, these proteins form a heterodimer that represses KNOXI genes *BP*, *KNAT2*, and *KNAT6* (Byrne *et al.*, 2000; Ori *et al.*, 2000; Semiarti *et al.*, 2001; Iwakawa *et al.*, 2002; Xu *et al.*, 2003; Guo *et al.*, 2008). AS1 and AS2 also specify adaxial fate, with a subtle influence on *A. thaliana* leaves but a more extreme contribution to adaxial fate in other species.

**Fig. 3.**
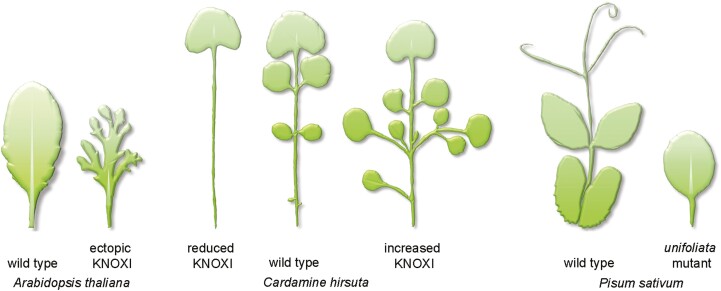
KNOXI is associated with changes in leaf shape complexity. Left: *Arabidopsis thaliana* wild-type simple leaf with undissected lamina. Ectopic expression of *STM* in the leaf results in leaf lobing. Middle: *Cardamine hirsuta* leaf reduced *ChSTM* has fewer leaflets compared with the wild-type compound leaf. *Cardamine hirsuta* leaf with overexpression of *ChSTM* has more leaflets compared with the wild type. Right: *Pisum sativum* wild-type compound leaf and *uni* mutant with a simple leaf. Leaf sizes are not to scale. Schematic diagrams of loss-of-function mutants and ectopic expression phenotypes are based on data in Hay and Tsiantis (2006), Demason *et al.* (2013), and Alvarez *et al.* (2016).

An interesting series of observations made many years ago in a number of plant species was that ectopic expression of KNOXI, via transgenes expressing KNOXI from ubiquitous, inducible, or leaf-specific promoters, resulted in changes of simple leaf shape to leaves with lobing of the margin (Fig. 3) (Sinha *et al.*, 1993; Lincoln *et al.*, 1994; Chuck *et al.*, 1996; Serikawa and Zambryski, 1997; Tamaoki *et al.*, 1997; Sakamoto *et al.*, 1999; Frugis *et al.*, 2001; Hay and Tsiantis, 2006). In *A. thaliana*, and in the simple leaves of tobacco and lettuce, the degree of lobing that results from the induction of ectopic KNOXI depends on dosage. Likewise, different KNOXI members show different degrees of leaf lobing (Sinha *et al.*, 1993; Chuck *et al.*, 1996; Tamaoki *et al.*, 1997; Hay *et al.*, 2003; Shani *et al.*, 2009). Elaboration of the leaf shape is proposed to be due to KNOXI activity delaying differentiation, thereby prolonging the window of growth permissive for patterning at the leaf margin (Shani *et al.*, 2009; Kierzkowski *et al.*, 2019).

**Fig. 4.**
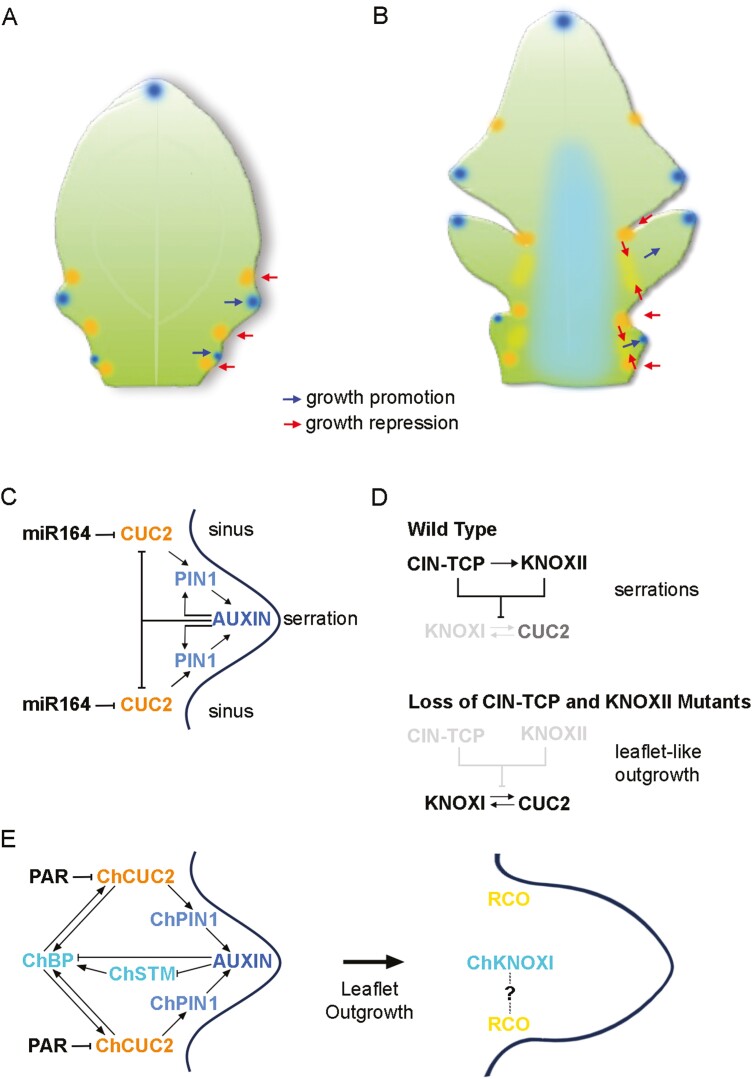
Growth patterning at the leaf margin. Schematic representations of young developing leaves of *A. thaliana* (A) and *C. hirsuta* (B). Growth along the leaf margin at serrations is promoted in regions of high auxin (dark blue) and is repressed in sinus regions where *CUC2* (orange) is expressed. In *C. hirsuta*, growth is also repressed at the base of leaflet outgrowths through the activity of RCO (yellow). In *C. hirsuta* (B), *ChSTM* (pale blue) has a broad expression domain and acts to delay differentiation. (C) At serrations in *A. thaliana*, a feedback loop promotes PIN1-mediated auxin peaks in serrations and CUC2 expression in sinus regions. (D) In wild-type *A. thaliana*, morphogenetic potential at leaf margins is reduced by CIN-TCP and KNOXII (black), which together repress expression of several KNOXI genes (light grey) and partially repress CUC2 (grey). In the absence of CIN-TCP and KNOXII (light grey), expression of several KNOXI genes is activated and CUC2 is up-regulated, leading to leaflet-like outgrowths. (E) In *C. hirsuta*, early leaflet outgrowth is promoted by a ChCUC2–auxin module that interacts with KNOXI genes. RCO is expressed at the base of developing leaflets. A regulatory interaction between RCO and KNOXI may or may not occur in *C. hirsuta.* Based on data in Bilsborough *et al.* (2011), Rast-Somssich *et al.* (2015), and Challa *et al.* (2021).

*Arabidopsis thaliana* leaves are spatulate with serrated margins. The serrations are small regularly distributed protuberances interspaced by sinuses. Despite being a rather obscure developmental feature, the study of genetic regulation of serrations has yielded interesting findings. Serrations become evident on the margin of young leaf primordia after the basic adaxial–abaxial, proximal–distal, and medial–lateral axes of growth and differentiation have been established (Nikovics *et al.*, 2006; Kawamura *et al.*, 2010; Bilsborough *et al.*, 2011; Jeon and Byrne, 2021). Essential genetic components of serration development are NAC-domain transcription factors, and in *A. thaliana* this includes CUP-SHAPED COYTYLEDON2 (CUC2) and CUC3. *CUC2* and *CUC3* are expressed in leaves and, along with the closely related gene *CUC1*, are also expressed in the shoot meristem. In the shoot meristem, these genes are restricted to a boundary region between organ primordia, where they repress growth and allow organ separation. They are required for meristem function and act in a positive feedback loop whereby CUC activates KNOXI gene expression and in turn STM activates *CUC* expression (Aida *et al.*, 1997, 1999; Vroemen *et al.*, 2003; Spinelli *et al.*, 2011; Scofield *et al.*, 2018). Within the leaf, *CUC2* is expressed in the sinus region between serrations and is required to form serrations (Fig. 4A) (Nikovics *et al.*, 2006; Bilsborough *et al.*, 2011). *CUC3* is partially redundant with *CUC2* and is involved in outgrowth of serrations, but is spatially more restricted and acts later in leaf development compared with *CUC2* (Hasson *et al.*, 2011; Serra and Perrot-Rechenmann, 2020). The level of CUC2 is an important determinant of the degree of marginal outgrowths. One mechanism of regulation is via the miRNA, miR164, which represses the level and domain of *CUC2* expression in leaf margins, and *CUC1* and *CUC2* in the shoot meristem (Laufs *et al.*, 2004; Mallory *et al.*, 2004; Nikovics *et al.*, 2006; Sieber *et al.*, 2007; Scofield *et al.*, 2018).

CUC2 works alongside the auxin efflux carrier PIN FORMED1 (PIN1) and the hormone auxin (Koenig *et al.*, 2009; Kawamura *et al.*, 2010; Bilsborough *et al.*, 2011). At early stages of leaf development, polar localization of PIN1 in leaf margin cells directs auxin to the distal tip of the leaf. As the leaf grows, the intracellular polar localization of PIN1 directs auxin towards recurrent convergence points along the leaf margin (Fig. 4A, C). Accumulation of auxin at these convergence points leads to tissue outgrowth (Hay *et al.*, 2006; Scarpella *et al.*, 2006; Kawamura *et al.*, 2010; Bilsborough *et al.*, 2011). CUC2 promotes PIN1 intracellular re-localization and is required to generate the auxin convergence points. Furthermore, auxin represses CUC2, thereby restricting expression to regions between outgrowths (Bilsborough *et al.*, 2011). Altering the CUC2–PIN1–auxin genetic module results in notable leaf phenotypes. Loss of PIN1 or CUC2 leads to loss of serrations and smooth leaf margins, whereas increasing CUC2 or disrupting auxin signalling leads to highly lobed leaves that show ectopic expression of the KNOXI gene *BP* (Nikovics *et al.*, 2006; Berger *et al.*, 2009; Bilsborough *et al.*, 2011). Thus KNOXI are not expressed in the *A. thaliana* leaf but this species has the genetic circuity for shaping a lobed leaf margin if KNOXI is provided (Hay *et al.*, 2006; Nikovics *et al.*, 2006; Hasson *et al.*, 2011).

KNOXII genes, on the other hand, are expressed in leaves (Fig. 1C). Interestingly these genes act in the opposite manner to KNOXI, promoting leaf maturation and a simple leaf shape. There is considerable redundancy between KNOXII genes. Mutations in individual genes have no or minimal phenotypic effects on leaf development. However, progressive loss of function of the KNOXII genes *KNAT3*, *KNAT4*, and *KNAT5* results in highly lobed leaves in a dosage-dependent manner (Furumizu *et al.*, 2015; Challa *et al.*, 2021). Furthermore, the BLH paralogues *SAW1* and *SAW2* are expressed in the leaf and redundantly suppress serrations (Fig. 1D). Mutations in either *SAW1* or *SAW2* have little or no phenotypic effect, but double mutants have more prominent serrations compared with the wild type. Mutations in either *SAW1* or *KNAT3* have only subtle enhancement of leaf serrations, but more prominent serrations occur in plants lacking both *SAW1* and *KNAT3*, consistent with functional heterodimer formation between these proteins (Kumar *et al.*, 2007; Furumizu *et al.*, 2015; H. Yu *et al.*, 2020; Jeon and Byrne, 2021). Similarly KNOXI–BLH interactions appear to determine leaf shape differences in cultivated varieties of lettuce. In lettuce, the KNOXI gene *LsKN1* and the BLH gene *LsSAW1* are associated with leaf shape differences, including a serrated or wavy leaf phenotype in heading varieties compared with non-heading varieties. A CACTA transposon insertion mediates higher levels of *LsKN1* transcription in heading varieties. This mutant allele probably arose after the first domestication of lettuce (C.  Yu *et al.*, 2020; Jia *et al.*, 2022). Heading varieties also have a loss-of-function mutation in *LsSAW1*. LsKN1 and LsSAW1 can form heterodimers which may inhibit activity as individually they act antagonistically in regulation of downstream targets (An *et al.*, 2022).

KNOXII appear to limit margin growth and promote maturation together with members of the CINCINNATA-TEOSINTE BRANCHED1/CYCLOIDEA/PCF (CIN-TCP) class of genes. Down-regulation of the three KNOXII genes *KNAT3*, *KNAT4*, and *KNAT5* or the five CIN-TCP genes, *TCP2*, *TCP3*, *TCP4*, *TCP10*, and *TCP24*, that are targeted by miR319 results in deeper marginal serrations (Palatnik *et al.*, 2003; Alvarez *et al.*, 2016; Challa *et al.*, 2021). However, reduced levels of both these KNOXII and CIN-TCP genes result in a highly ramified leaf lamina with distinct leaflet-like outgrowths, and outgrowths upon outgrowths. This phenotype requires the CUC2–auxin module and is associated with ectopic expression of KNOXI genes *KNAT2* and *KNAT6* (Fig. 4D) (H. Yu *et al.*, 2020; Challa *et al.*, 2021). TCP4 directly activates the KNOXII genes *KNAT3* and *KNAT4*, while TCP5, which is not a target of miR319, also activates expression of *KNAT3* as well as *SAW1* (H.  Yu *et al.*, 2020; Challa *et al.*, 2021). An emerging theme is one where the maturation status within the developing leaf is controlled by members of several different transcription factor classes, and considerable genetic redundancy serves to control marginal blastozone activity.

## *Brassicaceae* species with lobed or compound leaves

In plants with simple leaves, KNOXI are typically not expressed in the leaf. In contrast KNOXI genes are expressed in the leaves of many angiosperms with compound leaves, although there are exceptions within the *Fabaceae*, as discussed below. As we shall see, the expression of KNOXI in compound leaves is a key driver of their complexity.

*Arabidopsis* species other than *A. thaliana* tend to have lobed leaves, which is predicted to be the ancestral shape within this plant genus, and multiple independent events have led to a derived simple leaf trait in several lineages (Piazza *et al.*, 2010). The KNOXI gene *STM* is expressed in the leaves of *A. lyrata*, *A. suecica*, *A. helleri*, and *Olimarabidopsis pumila*. In *A. suecica*, RNAi-mediated silencing of *STM* reduces leaf lobing. In addition, interspecies crosses show that lobing is a dominant trait associated with higher transcription of the *STM* allele from *A. lyrata* and *A. helleri* relative to *A. thaliana. STM* has probably been a target for evolution of leaf shape. Potentially the simple leaf of *A. thaliana* evolved from an ancestor with lobed leaves through positive selection for changes in the promoter of *STM* that led to loss of expression in the leaf (Piazza *et al.*, 2010).

*Cardamine hirsuta* is close relative of Arabidopsis within the *Brassicaceae* (Hay and Tsiantis, 2006; Hay *et al.*, 2014; Nikolov *et al.*, 2019; Hendriks *et al.*, 2023). *Cardamine hirsuta* has pinnately compound leaves, with each leaf comprising a rachis with lateral leaflets and a terminal leaflet. The leaflet lamina is borne on a short stalk or petiolule attached to the rachis (Fig. 2). *Cardamine hirsuta* has provided key insights into the genetic and potential evolutionary changes required to transition between simple and compound leaf shapes. As in *A. thaliana*, KNOXI are down-regulated in the initiating leaf primordium of *C. hirsuta*, but in this species KNOXI expression is reactivated in developing leaves. This reactivation is required to produce leaflets. *ChSTM*, the orthologue of *STM*, has a central function in production of leaflets since reducing *ChSTM* results in loss of leaflets and simplifies the leaf (Fig. 3). On the other hand, loss of *ChBP* does not reduce leaflets, due to redundancy with *ChSTM* and possibly also redundancy with *ChKNAT2* and *ChKNAT6* (Hay and Tsiantis, 2006; Rast-Somssich *et al.*, 2015). Interspecies transgene expression studies, where *A. thaliana* genes are expressed in *C. hirsuta* and *C. hirsuta* genes are expressed in *A. thaliana*, demonstrate that promoter regions drive gene expression in a pattern reflecting the species of origin. This tells us firstly, that *cis*-acting elements in promoter regions of *STM* and *BP* are responsible for the species differences in expression patterns and, secondly, that both species have all the necessary *trans*-acting elements for appropriate expression (Hay and Tsiantis, 2006). *ChSTM* and *ChBP* are expressed on the abaxial and adaxial sides of the leaf, respectively. This difference may, to some extent, account for their different contributions to leaf shape, although ChSTM and ChBP proteins also differ in their capacity to induce leaf development changes in *A. thaliana* (Hay and Tsiantis, 2006; Rast-Somssich *et al.*, 2015).

The distinct contributions of *ChSTM* and *ChBP* to leaf development are further highlighted through their response to CUC genes. As in *A. thaliana*, a CUC–auxin genetic module is active in generating outgrowths along the *C. hirsuta* leaf margin (Hay and Tsiantis, 2006; Barkoulas *et al.*, 2008; Blein *et al.*, 2008). Leaflet initiation is associated with a peak of auxin along the margin of the rachis, and this requires ChPIN1. Disrupting auxin distribution through mutations in *ChPIN1* or treatment with auxin produces few leaflets or ectopic leaf lamina in regions between leaflets, respectively. The regulated distribution of auxin along the leaf margin therefore promotes leaflet outgrowth and separation (Barkoulas *et al.*, 2008). *ChCUC2* and *ChCUC*3 expression in *C. hirsuta*, and *CUC3* homologues in other compound leaf species, are restricted to the proximal and distal boundary of leaflets. *ChCUC2* is regulated by a miR164 encoded by the *PARSLEY* (*PAR*) gene (Rast-Somssich *et al.*, 2015). Overexpressing miR164 or down-regulation of *ChCUC2* leads to fewer leaflets and to leaflet fusions. By contrast, *par* mutants have more leaflets and this is associated with increased *ChBP* but not *ChSTM*. Indeed, in the *C. hirsuta* leaf, *ChSTM* appears to regulate *ChBP* (Fig. 4E) (Blein *et al.*, 2008; Berger *et al.*, 2009; Rast-Somssich *et al.*, 2015). At the leaf margin *ChSTM* is expressed in a pattern complementary to PIN1 and auxin peaks, and *ChSTM* can be repressed by synthetic auxin. In addition, increasing KNOXI promotes expression of *C. hirsuta CUC* genes (Barkoulas *et al.*, 2008; Blein *et al.*, 2008). A CUC–auxin genetic feedback module therefore regulates serrations in *A. thaliana* and leaflets in *C. hirsuta*. In *C. hirsuta* this regulatory module interacts with KNOXI to control leaflet development (Rast-Somssich *et al.*, 2015). Interestingly, *ChBP* is localized to the base of leaflets in a region overlapping with *ChCUC2*. While KNOXI act as global repressors of differentiation in the leaf, more restricted spatial expression may direct the formation of boundaries between regions of active and repressed growth (Rast-Somssich *et al.*, 2015; Challa *et al.*, 2021).

The CUC–auxin module and KNOXI account for regions of growth and repression at the margin of the rachis in the *C. hirsuta* compound leaf, but they do not account for the shape of leaflets. This requires the growth repressor REDUCED COMPLEXITY (RCO). RCO is necessary for development of leaflets as loss-of-function mutations result in a lobed leaf (Vlad *et al.*, 2014). KNOXI are expressed relatively broadly and act as global regulators of growth, delaying differentiation and thereby providing an extended morphogenic window for activity of local leaf margin patterning genes. On the other hand, *RCO* expression is restricted to the base of marginal outgrowths in a region nearly complementary to that of *ChCUC2*. RCO function is largely spatially and temporally separated from that of ChCUC2 and locally represses growth after ChCUC2–auxin-mediated patterning of the leaf margin (Fig. 4B, E) (Kierzkowski *et al.*, 2019; Bhatia *et al.*, 2023). This implies that there is no direct interaction between RCO and the ChCUC2–auxin module. Instead, RCO acts in the context of patterning set up by ChCUC2 and auxin.

Comparatively, serrations and leaflets are shaped through differential anisotropic growth in regions of high auxin peaks, with KNOXI delaying differentiation and permitting a long duration of outgrowth for leaflets relative to serrations (Kierzkowski *et al.*, 2019). Two additional features distinguish serrations and leaflets. Firstly, serrations are formed at the margin of leaf lamina, whereas leaflets are formed at the margin of the rachis. Thus the outgrowths initiate in different morphological and cellular contexts. Secondly, confined expression of *RCO* along the base of outgrowths uniquely serves to restrict growth in the proximal zone and along the mediolateral axis of the leaflet to generate a narrow petiolule (Kierzkowski *et al.*, 2019; Wang *et al.*, 2022). Remarkably KNOXI or *RCO* expression in *A. thaliana* generates a lobed leaf, but a compound leaf with distinct leaflets can be generated through combined expression of KNOXI and *RCO* (Chang *et al.*, 2019; Kierzkowski *et al.*, 2019). Localized expression of an *RCO* transgene to the base of outgrowths in *A. thaliana* partly requires loss of KNOXI repressors, *AS1* or *AS2*, suggesting that in this heterologous context there is subtle signalling between RCO and KNOXI growth regulators (Wang *et al.*, 2022). However, the precise relationship between KNOXI and *RCO* in *C. hirsuta* is still to be determined.

RCO is a homeodomain protein related to LATE MERISTEM IDENTITY 1 (LMI1). The *LMI1* genes interestingly have undergone duplications and gene loss within different lineages of the *Brassicaceae*. *RCO* is one of three tandem *LMI1* genes in *C. hirsuta*, *Capsella grandiflora*, *Capsella rubella*, and *A. lyrata. RCO* is not present in the *A. thaliana* genome, which has only one *LMI1* gene (Sicard *et al.*, 2014; Vlad *et al.*, 2014). Different LMI1 class proteins from various species induce lobed leaves to some extent in *A. thaliana* when driven by the *ChRCO* promoter. As such, a unique property of *RCO* relative to other *LMI1* genes is the spatial expression pattern at the base of the leaflet (Vlad *et al.*, 2014; Vuolo *et al.*, 2016). RCO expression levels also account for leaf shape differences between the *Capsella* species *C. grandiflora*, which has a simple leaf shape, and *C. rubella*, which has lobed leaves. A single major quantitative trait locus (QTL) contributes to this phenotypic difference whereby the *C. rubella RCO* allele is more highly expressed than the *RCO* allele of *C. grandiflora* (Sicard *et al.*, 2014). Likewise leaf shape in different cotton species (*Gossypium* sp.) varies from entire leaves lacking dissection, to deeply lobed leaves. In cultivars of *G. hirsutum*, different leaf lobing is largely due to the *okra* locus, which encodes an *LMI1* homologue. Large lobes in okra leaves and severe leaf lobing in super-okra leaves is associated with higher levels of a full-length *okra* gene, whereas mild and intermediate-lobing cultivars have a mutation in *okra* leading to a truncated protein. *LMI1*-associated changes in leaf shape in cotton therefore appear to involve both transcription and protein activity (Zhu *et al.*, 2016; Andres *et al.*, 2017).

## Beyond *Brassicaceae*: other eudicots with compound leaves

The ancestral leaf form in angiosperms is predicted to be a simple shape with compound leaves, and reversion from compound to simple leaf shape occurring multiple independent times. This raises the question as to whether the same or different genetic pathways control leaf shape complexity throughout angiosperms (Bharathan *et al.*, 2002; Groot *et al.*, 2005; Champagne *et al.*, 2007; Sousa-Baena *et al.*, 2014). The study of compound leaf development in several species has provided significant insights into which genetic pathways are conserved, and the pathways that differ between species. Beyond the *Brassicaceae*, well-studied species with compound leaves are *Solanum lycopersicum* (tomato), *Pisum sativum* (pea), *Medicago truncatula* (barrel clover), and *Vigna radiata* (mung bean). These species each offer unique opportunities to understand the genetic regulation and evolution of leaf shape diversity.

Wild-type or cultivated tomato has a bipinnately compound leaf composed of a terminal leaflet and three to four pairs of primary leaflets that themselves have secondary leaflets. Additionally, the leaflet margins are lobed, adding to shape complexity (Fig. 2). In these respects, tomato allows examination of whether the context and extent of margin patterning of the leaf lamina are regulated by the same genetic pathways or whether they are developmentally independent. Certainly, KNOXI proteins extend the timing of meristematic activity at the leaf margin, and their level of expression determines the degree of leaflet ramification. Reducing KNOXI levels results in a simplified leaf, whereas mutations or transgenes that increase KNOXI produce a leaf with a higher order of leaflets (Sinha *et al.*, 1993; Hareven *et al.*, 1996; Chen *et al.*, 1997; Janssen *et al.*, 1998; Shani *et al.*, 2009).

Several interesting examples of changes to TALE homeobox gene expression and leaf shape diversity in wild and heirloom cultivars of tomato have been described. The Russian heirloom variety, Silvery Fir Tree (SiFT) generates more leaflets compared with standard cultivars. This phenotype is due to a nucleotide polymorphism that disrupts *BIPINNATA* (*BIP*), an orthologue of the *A. thaliana* BLH class genes *SAW1* and *SAW2*. BIP and SAW repress KNOXI in the leaf (Kumar *et al.*, 2007; Kimura *et al.*, 2008; Nakayama *et al.*, 2021). The second example relates to distinct wild populations of tomato on the Galapagos Islands that have different leaf complexity. *Solanum cheesmaniae* has a single order of leaflets whereas *S. galapagense* has three orders of leaflets. The mutation responsible for the increased leaf dissection in *S. galapagense* is a single nucleotide change in the promoter region of the gene *PETROSELINUM* (*PTS*) [also known as *TOMATO KNOX-LIKE HOMEODOMAIN PROTEIN 1* (*TDK1*)]. *PTS* is a class M KNOX gene, encoding a MEINOX domain but not a homeodomain. *PTS* is up-regulated in *S. galapagense* compared with *S. cheesmaniae*, and this higher level of expression influences KNOXI activity. The KNOXI gene *TOMATO KNOTTED-1* (*TKN1*) is transcriptionally up-regulated in *S. galapagense* and in plants carrying the *S. galapagense pts* allele. In addition, PTS disrupts the *in vitro* protein–protein interaction between BIP and the KNOXI class protein LeT6. PTS therefore appears to regulate KNOXI activity in the leaf both transcriptionally and post-transcriptionally (Kimura *et al.*, 2008; Nakayama *et al.*, 2021). In addition to these two examples, gene regulatory network analysis indicates that levels of the transcription factor BLADE-ON-PETIOLE (BOP) correlate with variation between three species of tomato, the cultivated *S. lycopersicum*, which has intermediate leaf complexity, and two wild relatives, *S. pennellii* with low leaf complexity and *S. habrochaites* with high leaf complexity. *Solanum pennellii* has higher levels of BOP relative to *S. lycopersicum*, which has higher levels than *S. habrochaites*. BOP regulates transcription of *PTS* and BLH class genes (Ichihashi *et al.*, 2014). Consistent with these examples where leaf shape is dependent on the regulation of KNOXI, an unbiased screen for leaflet formation in *A. thaliana* carrying the *C. hirsuta RCO* gene only identified mutations in *AS1* and *AS2*, two repressors of KNOXI (Wang *et al.*, 2022). The evolution of complex leaves in tomato and Arabidopsis species may therefore involve modifying KNOXI expression, through change in the promoter region of KNOXI genes that alter their expression, or change in components of the network regulating KNOXI expression or activity (Hay and Tsiantis, 2006; Ichihashi *et al.*, 2014; Wang *et al.*, 2022).

Leaflet production and separation as well as lobing of leaflets in tomato involves GOBLET (GOB), a transcription factor closely related to CUC2 (Berger *et al.*, 2009). Loss-of-function mutations in GOB lead to simplified leaves with only primary, unlobed leaflets. On the other hand, GOB dominant mutants, which have increased expression due to a mutation in a miR164-binding site, have deeply lobed leaflets and fewer leaflets (Berger *et al.*, 2009; Israeli *et al.*, 2021). GOB therefore appears to have context-dependent effects. At the margin of the leaflet lamina, GOB regulates the extent of lobing, whereas along the rachis GOB regulates production of leaflets. In addition to GOB, multiple auxin response regulators are required for leaflet production. These include the indole acetic acid (Aux/IAA) protein ENTIRE (E) and members of the AUXIN RESPONSE FACTOR (ARF) class such as SlMP/SlARF5. Aux/IAA proteins repress the activity of ARF proteins, but this repression is released in the presence of high auxin where Aux/IAA is degraded. *E* is expressed in the intercalary region between leaflets, and ARFs are expressed within the outgrowing leaflet. Both GOB and SlMP are required for the increased leaflet ramification observed in plants with elevated expression of KNOXI (Berger *et al.*, 2009; Koenig *et al.*, 2009; Ben-Gera *et al.*, 2012; Israeli *et al.*, 2019, 2021; Xiong and Jiao, 2019). The tomato compound leaf shape conferred by KNOXI is also dependent on CIN-TCP class transcription factors such as Lanceolate (LA). Although *LA* dominant mutants have a simple leaf and suppress the highly ramified leaf phenotype induced by overexpression of KNOXI, reducing levels of LA promotes leaflet production in a manner that is additive with overexpression of KNOXI. LA may act independently of KNOXI but provide a permissive developmental environment for KNOXI activity (Ori *et al.*, 2007).

The value of studying leaf development across a broad spectrum of the plant phylogenetic tree is aptly demonstrated by key findings on leaf development in compound leaves of species in the *Fabaceae*, commonly known as the legumes. Typically, these species have pinnately compound leaves with lateral leaflets and a terminal leaflet, each connected to a central rachis, although in some species leaves are trifoliolate or palmate. Further variations such as in *P. sativum*, the common garden pea, include prominent leaf-like stipules and reduction of leaflets into slender tendrils (Fig. 2).

Pea is a member of a large subclade of legumes, the inverted repeat-lacking clade (IRLC), characterized by a loss of one copy of a chloroplast genome inverted repeat sequence. *Fabaceae* species outside the IRLC clade, including *Glycine max* (soybean), *Mimosa pudica*, *Acacia hindsii*, *Phaseolus vulgaris* (bean), and *Lotus japonicus*, have compound leaves with KNOXI expression in the leaf, and in this respect are similar to *C. hirsuta* and tomato (Champagne *et al.*, 2007). Conversely KNOXI genes are typically not detected in pea or *M. truncatula* leaves or in leaves of other IRLC species *M. sativa* (alfalfa), *Vigna radiata* (mung bean), *Wisteria sinensis* (wisteria), and *Vicia faba* (fava bean) (Gourlay *et al.*, 2000; Hofer *et al.*, 2001; Champagne *et al.*, 2007; Ge *et al.*, 2014; Zhou *et al.*, 2014). Compound leaf development is instead dependent on transcription factor genes related to *UNIFOLIATA* (*UNI*) in pea and *SINGLE LEAFLET1* (*SGL1*) in *M. truncatula*. Mutations in these genes have fewer lateral leaflets and may develop as a simple leaf (Fig. 3) (Hofer *et al.*, 1997; Wang *et al.*, 2008). UNI and SGL1 are orthologues of the transcription factors FLORICAULA (FLO) in *Antirrhinum* and LEAFY (LFY) in *A. thaliana*, which control floral meristem identity (Moyroud *et al.*, 2010). Although UNI and SGL1 appear to be the principal drivers of leaf complexity in these species, in pea, *M. truncatula*, and *M. sativa*, mutations in orthologues of *AS1* result in changes in leaf development and some ectopic KNOXI expression in leaves. The phenotypic consequences vary somewhat but include ectopic lamina, more prominent leaflet serrations, and more leaflets (Tattersall *et al.*, 2005; Champagne *et al.*, 2007; Ge *et al.*, 2014; Zhou *et al.*, 2014). These are relatively subtle phenotypes, possibly due to modest levels of KNOXI in these mutants since transgene-induced high levels of KNOXI in leaves of *M. truncatula* induce variations in leaflet number and higher order leaflets (Zhou *et al.*, 2014). Surprisingly, *STM* from *A. thaliana* can rescue the *sgl1* mutant leaf phenotype, suggesting that both LFY and KNOXI class genes have common downstream targets (Pautot *et al.*, 2022). In the non-IRLC species tomato, *G. max*, *L. japonicus*, and *V. radiata*, KNOXI are detected in the leaves but LFY orthologues also play a minor role in promoting production of leaflets (Molinero-Rosales *et al.*, 1999; Dong *et al.*, 2005; Champagne *et al.*, 2007; Jiao *et al.*, 2019). As such, LFY-related genes and KNOXI may have parallel functions or have shared downstream targets. From an evolutionary point of view, in the IRLC clade *Fabaceae* species both pathways may have been functional. The loss of a role for KNOX1 genes in leaf development in these species appears to have occurred via loss of expression in the leaf. This may have occurred through changes in *cis*-regulatory sequences of KNOX1 genes or could be the result of changes to upstream regulators of KNOX1 genes.

Interestingly the CUC–auxin genetic module appears to have a conserved function in leaflet development in IRLC species, but this module interfaces with UNI rather than KNOXI. In pea, as in *C. hirsuta* and tomato, *CUC3* genes are expressed in the proximal and distal boundary of leaflets, and peaks of auxin occur where leaflets initiate. Disrupting *CUC3* and auxin leads to a more simplified leaf (Blein *et al.*, 2008; Demason *et al.*, 2013). Likewise, in *M. truncatula*, mutations of *SMOOTH LEAF MARGIN1* (*SLM1*), an orthologue of PIN1, result in a loss of leaf serrations and fewer lateral leaflets. However, in *sml1* mutants there are additional terminal leaflets, suggesting that different mechanisms or different degrees of gene redundancy regulate *M. truncatula* lateral and terminal leaflets (Zhou *et al.*, 2011). Mutations in a *CUC3*-related gene, *MtNAM*, result in fusion of leaflets but do not affect leaflet initiation or margin serrations possibly due to gene redundancy (Cheng *et al.*, 2012). Although CUC–auxin represents a common genetic module for serrations, lobes, and leaflets in many plant species, the spatial and temporal activity of this pathway appears to depend on genetic redundancy of CUC class genes.

## Conclusions

As it is theorized that compound leaves are the ancestral leaf form of many angiosperm lineages, with multiple incidences of reversion to simple leaves, we must question just how conserved leaf developmental pathways are. In this context, TALE homeodomain proteins present an enduring point of interest as their continuity between species and their function in leaf shape complexity allow a useful starting point to probe the evolution of leaf regulatory modules.

Several themes begin to emerge when discussing the importance of TALE homeodomain proteins in leaf development, which to date has largely focused on KNOXI proteins. Firstly, whilst KNOXI are conserved across land plant taxa, an expansion of this gene class has occurred throughout evolutionary history. Such changes result in gene redundancies that are predicted to have lent plasticity to the regulatory modules that underpin leaf development. In turn, this plasticity may have broadened the potential protein–protein interactions that can interface with established genetic regulatory networks. Likewise, changes to KNOXI expression domains through alterations in promoter regions are, in some cases, sufficient to generate morphological variation in leaves. These perturbations to regulatory mechanisms of development account for some of the diverse range of leaf forms observable in the natural world today. From here, we can more broadly assess the types of changes leading to natural variation in leaf shape and whether the same regulatory modules are the target for evolutionary change. Several plant species undergo radical changes in leaf shape in response to environmental influences of temperature, light quality, and in response to aquatic or terrestrial conditions (Chitwood and Sinha, 2016). Whether TALE homeodomain transcription factor-mediated changes in leaf shape have adaptive significance remains to be uncovered.

## References

[CIT0001] Aida M , Ishida T, Fukaki H, Fujisawa H, Tasaka M. 1997. Genes involved in organ separation in Arabidopsis: an analysis of the *cup-shaped cotyledon* mutant. The Plant Cell 9, 841–857. 9212461 10.1105/tpc.9.6.841 PMC156962

[CIT0002] Aida M , Ishida T, Tasaka M. 1999. Shoot apical meristem and cotyledon formation during Arabidopsis embryogenesis: interaction among the *CUP-SHAPED COTYLEDON* and *SHOOT MERISTEMLESS* genes. Development 126, 1563–1570. 10079219 10.1242/dev.126.8.1563

[CIT0003] Alvarez JP , Furumizu C, Efroni I, Eshed Y, Bowman JL. 2016. Active suppression of a leaf meristem orchestrates determinate leaf growth. eLife 5, e15023. 27710768 10.7554/eLife.15023 PMC5096885

[CIT0004] An G , Yu C, Yan C, et al. 2022. Loss-of-function of *SAWTOOTH 1* affects leaf dorsiventrality genes to promote leafy heads in lettuce. The Plant Cell 34, 4329–4347. 35916734 10.1093/plcell/koac234 PMC9614500

[CIT0005] Andres RJ , Coneva V, Frank MH, et al. 2017. Modifications to a *LATE MERISTEM IDENTITY1* gene are responsible for the major leaf shapes of Upland cotton (*Gossypium hirsutum* L.). Proceedings of the National Academy of Sciences, USA 114, E57–E66. 10.1073/pnas.1613593114 PMC5224360 27999177

[CIT0006] Arun A , Coelho SM, Peters AF, et al. 2019. Convergent recruitment of TALE homeodomain life cycle regulators to direct sporophyte development in land plants and brown algae. eLife 8, e43101. 30644818 10.7554/eLife.43101 PMC6368402

[CIT0007] Barkoulas M , Hay A, Kougioumoutzi E, Tsiantis M. 2008. A developmental framework for dissected leaf formation in the *Arabidopsis* relative *Cardamine hirsuta*. Nature Genetics 40, 1136–1141. 19165928 10.1038/ng.189

[CIT0008] Becker A , Bey M, Burglin TR, Saedler H, Theissen G. 2002. Ancestry and diversity of *BEL1*-like homeobox genes revealed by gymnosperm (*Gnetum gnemon*) homologs. Development Genes and Evolution 212, 452–457. 12373591 10.1007/s00427-002-0259-7

[CIT0009] Bellaoui M , Pidkowich MS, Samach A, Kushalappa K, Kohalmi SE, Modrusan Z, Crosby WL, Haughn GW. 2001. The Arabidopsis BELL1 and KNOX TALE homeodomain proteins interact through a domain conserved between plants and animals. The Plant Cell 13, 2455–2470. 11701881 10.1105/tpc.010161 PMC139464

[CIT0010] Belles-Boix E , Hamant O, Witiak SM, Morin H, Traas J, Pautot V. 2006. *KNAT6*: an Arabidopsis homeobox gene involved in meristem activity and organ separation. The Plant Cell 18, 1900–1907. 16798887 10.1105/tpc.106.041988 PMC1533965

[CIT0011] Ben-Gera H , Shwartz I, Shao MR, Shani E, Estelle M, Ori N. 2012. ENTIRE and GOBLET promote leaflet development in tomato by modulating auxin response. The Plant Journal 70, 903–915. 22332729 10.1111/j.1365-313X.2012.04939.x

[CIT0012] Berger Y , Harpaz-Saad S, Brand A, Melnik H, Sirding N, Alvarez JP, Zinder M, Samach A, Eshed Y, Ori N. 2009. The NAC-domain transcription factor GOBLET specifies leaflet boundaries in compound tomato leaves. Development 136, 823–832. 19176589 10.1242/dev.031625

[CIT0013] Bertolino E , Reimund B, Wildt-Perinic D, Clerc RG. 1995. A novel homeobox protein which recognizes a TGT core and functionally interferes with a retinoid-responsive motif. Journal of Biological Chemistry 270, 31178–31188. 8537382 10.1074/jbc.270.52.31178

[CIT0014] Bharathan G , Goliber TE, Moore C, Kessler S, Pham T, Sinha NR. 2002. Homologies in leaf form inferred from *KNOXI* gene expression during development. Science 296, 1858–1860. 12052958 10.1126/science.1070343

[CIT0015] Bharathan G , Janssen BJ, Kellogg EA, Sinha N. 1997. Did homeodomain proteins duplicate before the origin of angiosperms, fungi, and metazoa? Proceedings of the National Academy of Sciences, USA 94, 13749–13753. 10.1073/pnas.94.25.13749 PMC28378 9391098

[CIT0016] Bhatia N , Wilson-Sanchez D, Strauss S, Vuolo F, Pieper B, Hu Z, Rambaud-Lavigne L, Tsiantis M. 2023. Interspersed expression of *CUP-SHAPED COTYLEDON2* and *REDUCED COMPLEXITY* shapes *Cardamine hirsuta* complex leaf form. Current Biology 33, 2977–2987. 37453425 10.1016/j.cub.2023.06.037

[CIT0017] Bhatt AM , Etchells JP, Canales C, Lagodienko A, Dickinson H. 2004. VAAMANA—a BEL1-like homeodomain protein, interacts with KNOX proteins BP and STM and regulates inflorescence stem growth in *Arabidopsis*. Gene 328, 103–111. 15019989 10.1016/j.gene.2003.12.033

[CIT0018] Bilsborough GD , Runions A, Barkoulas M, Jenkins HW, Hasson A, Galinha C, Laufs P, Hay A, Prusinkiewicz P, Tsiantis M. 2011. Model for the regulation of *Arabidopsis thaliana* leaf margin development. Proceedings of the National Academy of Sciences, USA 108, 3424–3429. 10.1073/pnas.1015162108 PMC3044365 21300866

[CIT0019] Blein T , Pulido A, Vialette-Guiraud A, Nikovics K, Morin H, Hay A, Johansen IE, Tsiantis M, Laufs P. 2008. A conserved molecular framework for compound leaf development. Science 322, 1835–1839. 19095941 10.1126/science.1166168

[CIT0020] Bowman JL , Sakakibara K, Furumizu C, Dierschke T. 2016. Evolution in the cycles of life. Annual Review of Genetics 50, 133–154. 10.1146/annurev-genet-120215-035227 27617970

[CIT0021] Bürglin TR. 1997. Analysis of TALE superclass homeobox genes (MEIS, PBC, KNOX, Iroquois, TGIF) reveals a novel domain conserved between plants and animals. Nucleic Acids Research 25, 4173–4180. 9336443 10.1093/nar/25.21.4173 PMC147054

[CIT0022] Bürglin TR. 1998. The PBC domain contains a MEINOX domain: coevolution of Hox and TALE homeobox genes? Development Genes and Evolution 208, 113–116. 9569353 10.1007/s004270050161

[CIT0023] Byrne ME , Barley R, Curtis M, Arroyo JM, Dunham M, Hudson A, Martienssen RA. 2000. *Asymmetric leaves1* mediates leaf patterning and stem cell function in *Arabidopsis*. Nature 408, 967–971. 11140682 10.1038/35050091

[CIT0024] Byrne ME , Groover AT, Fontana JR, Martienssen RA. 2003. Phyllotactic pattern and stem cell fate are determined by the Arabidopsis homeobox gene *BELLRINGER*. Development 130, 3941–3950. 12874117 10.1242/dev.00620

[CIT0025] Byrne ME , Simorowski J, Martienssen RA. 2002. *ASYMMETRIC LEAVES1* reveals knox gene redundancy in *Arabidopsis*. Development 129, 1957–1965. 11934861 10.1242/dev.129.8.1957

[CIT0026] Challa KR , Rath M, Sharma AN, Bajpai AK, Davuluri S, Acharya KK, Nath U. 2021. Active suppression of leaflet emergence as a mechanism of simple leaf development. Nature Plants 7, 1264–1275. 34312497 10.1038/s41477-021-00965-3

[CIT0027] Champagne CE , Goliber TE, Wojciechowski MF, Mei RW, Townsley BT, Wang K, Paz MM, Geeta R, Sinha NR. 2007. Compound leaf development and evolution in the legumes. The Plant Cell 19, 3369–3378. 17993625 10.1105/tpc.107.052886 PMC2174894

[CIT0028] Chang L , Mei G, Hu Y, Deng J, Zhang T. 2019. *LMI1-like* and *KNOX1* genes coordinately regulate plant leaf development in dicotyledons. Plant Molecular Biology 99, 449–460. 30689141 10.1007/s11103-019-00829-7

[CIT0029] Chen H , Rosin FM, Prat S, Hannapel DJ. 2003. Interacting transcription factors from the three-amino acid loop extension superclass regulate tuber formation. Plant Physiology 132, 1391–1404. 12857821 10.1104/pp.103.022434 PMC167079

[CIT0030] Chen JJ , Janssen BJ, Williams A, Sinha N. 1997. A gene fusion at a homeobox locus: alterations in leaf shape and implications for morphological evolution. The Plant Cell 9, 1289–1304. 9286107 10.1105/tpc.9.8.1289 PMC156998

[CIT0031] Cheng X , Peng J, Ma J, Tang Y, Chen R, Mysore KS, Wen J. 2012. *NO APICAL MERISTEM* (*MtNAM*) regulates floral organ identity and lateral organ separation in *Medicago truncatula*. New Phytologist 195, 71–84. 22530598 10.1111/j.1469-8137.2012.04147.x

[CIT0032] Chitwood DH , Sinha NR. 2016. Evolutionary and environmental forces sculpting leaf development. Current Biology 26, R297–R306. 27046820 10.1016/j.cub.2016.02.033

[CIT0033] Chuck G , Lincoln C, Hake S. 1996. *KNAT1* induces lobed leaves with ectopic meristems when overexpressed in Arabidopsis. The Plant Cell 8, 1277–1289. 8776897 10.1105/tpc.8.8.1277 PMC161241

[CIT0034] Cole M , Nolte C, Werr W. 2006. Nuclear import of the transcription factor SHOOT MERISTEMLESS depends on heterodimerization with BLH proteins expressed in discrete sub-domains of the shoot apical meristem of *Arabidopsis thaliana*. Nucleic Acids Research 34, 1281–1292. 16513846 10.1093/nar/gkl016 PMC1388269

[CIT0035] Demason DA , Chetty V, Barkawi LS, Liu X, Cohen JD. 2013. *UNIFOLIATA–AFILA* interactions in pea leaf morphogenesis. American Journal of Botany 100, 478–495. 23400494 10.3732/ajb.1200611

[CIT0036] Dong ZC , Zhao Z, Liu CW, Luo JH, Yang J, Huang WH, Hu XH, Wang TL, Luo D. 2005. Floral patterning in *Lotus japonicus*. Plant Physiology 137, 1272–1282. 15824286 10.1104/pp.104.054288 PMC1088320

[CIT0037] Ezura K , Nakamura A, Mitsuda N. 2022. Genome-wide characterization of the TALE homeodomain family and the KNOX–BLH interaction network in tomato. Plant Molecular Biology 109, 799–821. 35543849 10.1007/s11103-022-01277-6

[CIT0038] Frugis G , Giannino D, Mele G, Nicolodi C, Chiappetta A, Bitonti MB, Innocenti AM, Dewitte W, Van Onckelen H, Mariotti D. 2001. Overexpression of *KNAT1* in lettuce shifts leaf determinate growth to a shoot-like indeterminate growth associated with an accumulation of isopentenyl-type cytokinins. Plant Physiology 126, 1370–1380. 11500537 10.1104/pp.126.4.1370 PMC117138

[CIT0039] Furumizu C , Alvarez JP, Sakakibara K, Bowman JL. 2015. Antagonistic roles for KNOX1 and KNOX2 genes in patterning the land plant body plan following an ancient gene duplication. PLoS Genetics 11, e1004980. 25671434 10.1371/journal.pgen.1004980 PMC4335488

[CIT0040] Gao J , Yang X, Zhao W, Lang T, Samuelsson T. 2015. Evolution, diversification, and expression of KNOX proteins in plants. Frontiers in Plant Science 6, 882. 26557129 10.3389/fpls.2015.00882 PMC4617109

[CIT0041] Ge L , Peng J, Berbel A, Madueno F, Chen R. 2014. Regulation of compound leaf development by *PHANTASTICA* in *Medicago truncatula*. Plant Physiology 164, 216–228. 24218492 10.1104/pp.113.229914 PMC3875802

[CIT0042] Gourlay CW , Hofer JM, Ellis TH. 2000. Pea compound leaf architecture is regulated by interactions among the genes *UNIFOLIATA*, *COCHLEATA*, *AFILA, AND TENDRIL-LESS*. The Plant Cell 12, 1279–1294. 10948249 10.1105/tpc.12.8.1279 PMC149102

[CIT0043] Groot EP , Sinha N, Gleissberg S. 2005. Expression patterns of STM-like KNOX and histone H4 genes in shoot development of the dissected-leaved basal eudicot plants *Chelidonium majus* and *Eschscholzia californica* (Papaveraceae). Plant Molecular Biology 58, 317–331. 16021398 10.1007/s11103-005-4548-1

[CIT0044] Guo M , Thomas J, Collins G, Timmermans MC. 2008. Direct repression of *KNOX* loci by the ASYMMETRIC LEAVES1 complex of *Arabidopsis*. The Plant Cell 20, 48–58. 18203921 10.1105/tpc.107.056127 PMC2254922

[CIT0045] Hackbusch J , Richter K, Muller J, Salamini F, Uhrig JF. 2005. A central role of *Arabidopsis thaliana* ovate family proteins in networking and subcellular localization of 3-aa loop extension homeodomain proteins. Proceedings of the National Academy of Sciences, USA 102, 4908–4912. 10.1073/pnas.0501181102 PMC555730 15781858

[CIT0046] Hagemann W , Gleissberg S. 1996. Organogenetic capacity of leaves: the significance of marginal blastozones in angiosperms. Plant Systematics and Evolution 199, 121–152.

[CIT0047] Hareven D , Gutfinger T, Parnis A, Eshed Y, Lifschitz E. 1996. The making of a compound leaf: genetic manipulation of leaf architecture in tomato. Cell 84, 735–744. 8625411 10.1016/s0092-8674(00)81051-x

[CIT0048] Hasson A , Plessis A, Blein T, Adroher B, Grigg S, Tsiantis M, Boudaoud A, Damerval C, Laufs P. 2011. Evolution and diverse roles of the *CUP-SHAPED COTYLEDON* genes in *Arabidopsis* leaf development. The Plant Cell 23, 54–68. 21258003 10.1105/tpc.110.081448 PMC3051246

[CIT0049] Hay A , Barkoulas M, Tsiantis M. 2006. ASYMMETRIC LEAVES1 and auxin activities converge to repress BREVIPEDICELLUS expression and promote leaf development in Arabidopsis. Development 133, 3955–3961. 16971475 10.1242/dev.02545

[CIT0050] Hay A , Jackson D, Ori N, Hake S. 2003. Analysis of the competence to respond to KNOTTED1 activity in Arabidopsis leaves using a steroid induction system. Plant Physiology 131, 1671–1680. 12692326 10.1104/pp.102.017434 PMC166923

[CIT0051] Hay A , Tsiantis M. 2006. The genetic basis for differences in leaf form between *Arabidopsis thaliana* and its wild relative *Cardamine hirsuta*. Nature Genetics 38, 942–947. 16823378 10.1038/ng1835

[CIT0052] Hay AS , Pieper B, Cooke E, et al. 2014. *Cardamine hirsuta*: a versatile genetic system for comparative studies. The Plant Journal 78, 1–15. 24460550 10.1111/tpj.12447

[CIT0053] Heisler MG , Byrne ME. 2020. Progress in understanding the role of auxin in lateral organ development in plants. Current Opinion in Plant Biology 53, 73–79. 31785585 10.1016/j.pbi.2019.10.007

[CIT0054] Heisler MG , Ohno C, Das P, Sieber P, Reddy GV, Long JA, Meyerowitz EM. 2005. Patterns of auxin transport and gene expression during primordium development revealed by live imaging of the *Arabidopsis* inflorescence meristem. Current Biology 15, 1899–1911. 16271866 10.1016/j.cub.2005.09.052

[CIT0055] Hendriks KP , Kiefer C, Al-Shehbaz IA, et al. 2023. Global Brassicaceae phylogeny based on filtering of 1,000-gene dataset. Current Biology 33, 4052–4068. 37659415 10.1016/j.cub.2023.08.026

[CIT0056] Hisanaga T , Fujimoto S, Cui Y, Sato K, Sano R, Yamaoka S, Kohchi T, Berger F, Nakajima K. 2021. Deep evolutionary origin of gamete-directed zygote activation by KNOX/BELL transcription factors in green plants. eLife 10, e57090. 34579806 10.7554/eLife.57090 PMC8478417

[CIT0057] Hofer J , Gourlay C, Michael A, Ellis TH. 2001. Expression of a class 1 *knotted1*-like homeobox gene is down-regulated in pea compound leaf primordia. Plant Molecular Biology 45, 387–398. 11352458 10.1023/a:1010739812836

[CIT0058] Hofer J , Turner L, Hellens R, Ambrose M, Matthews P, Michael A, Ellis N. 1997. *UNIFOLIATA* regulates leaf and flower morphogenesis in pea. Current Biology 7, 581–587. 9259553 10.1016/s0960-9822(06)00257-0

[CIT0059] Ichihashi Y , Aguilar-Martinez JA, Farhi M, Chitwood DH, Kumar R, Millon LV, Peng J, Maloof JN, Sinha NR. 2014. Evolutionary developmental transcriptomics reveals a gene network module regulating interspecific diversity in plant leaf shape. Proceedings of the National Academy of Sciences, USA 111, E2616–E2621. 10.1073/pnas.1402835111 PMC4078850 24927584

[CIT0060] Israeli A , Ben-Herzel O, Burko Y, Shwartz I, Ben-Gera H, Harpaz-Saad S, Bar M, Efroni I, Ori N. 2021. Coordination of differentiation rate and local patterning in compound-leaf development. New Phytologist 229, 3558–3572. 33259078 10.1111/nph.17124

[CIT0061] Israeli A , Capua Y, Shwartz I, Tal L, Meir Z, Levy M, Bar M, Efroni I, Ori N. 2019. Multiple auxin-response regulators enable stability and variability in leaf development. Current Biology 29, 1746–1759. 31104930 10.1016/j.cub.2019.04.047

[CIT0062] Iwakawa H , Ueno Y, Semiarti E, et al. 2002. The *ASYMMETRIC LEAVES2* gene of *Arabidopsis thaliana*, required for formation of a symmetric flat leaf lamina, encodes a member of a novel family of proteins characterized by cysteine repeats and a leucine zipper. Plant and Cell Physiology 43, 467–478. 12040093 10.1093/pcp/pcf077

[CIT0063] Janssen BJ , Lund L, Sinha N. 1998. Overexpression of a homeobox gene, *LeT6*, reveals indeterminate features in the tomato compound leaf. Plant Physiology 117, 771–786. 9662520 10.1104/pp.117.3.771 PMC34932

[CIT0064] Jeon HW , Byrne ME. 2021. SAW homeodomain transcription factors regulate initiation of leaf margin serrations. Journal of Experimental Botany 72, 1738–1747. 33247922 10.1093/jxb/eraa554

[CIT0065] Jia P , Wang Y, Sharif R, Dong QL, Liu Y, Luan HA, Zhang XM, Guo SP, Qi GH. 2023. KNOTTED1-like homeobox (KNOX) transcription factors—hubs in a plethora of networks: a review. International Journal of Biological Macromolecules 253, 126878. 37703987 10.1016/j.ijbiomac.2023.126878

[CIT0066] Jia Y , Yu P, Shao W, An G, Chen J, Yu C, Kuang H. 2022. Up-regulation of *LsKN1* promotes cytokinin and suppresses gibberellin biosynthesis to generate wavy leaves in lettuce. Journal of Experimental Botany 73, 6615–6629. 35816166 10.1093/jxb/erac311

[CIT0067] Jiao K , Li X, Su S, Guo W, Guo Y, Guan Y, Hu Z, Shen Z, Luo D. 2019. Genetic control of compound leaf development in the mungbean (*Vigna radiata* L.). Horticulture Research 6, 23. 30729013 10.1038/s41438-018-0088-0 PMC6355865

[CIT0068] Kawamura E , Horiguchi G, Tsukaya H. 2010. Mechanisms of leaf tooth formation in Arabidopsis. The Plant Journal 62, 429–441. 20128880 10.1111/j.1365-313X.2010.04156.x

[CIT0069] Kerstetter R , Vollbrecht E, Lowe B, Veit B, Yamaguchi J, Hake S. 1994. Sequence analysis and expression patterns divide the maize *knotted1*-like homeobox genes into two classes. The Plant Cell 6, 1877–1887. 7866030 10.1105/tpc.6.12.1877 PMC160568

[CIT0070] Kierzkowski D , Runions A, Vuolo F, et al. 2019. A growth-based framework for leaf shape development and diversity. Cell 177, 1405–1418.e17. 31130379 10.1016/j.cell.2019.05.011 PMC6548024

[CIT0071] Kimura S , Koenig D, Kang J, Yoong FY, Sinha N. 2008. Natural variation in leaf morphology results from mutation of a novel *KNOX* gene. Current Biology 18, 672–677. 18424140 10.1016/j.cub.2008.04.008

[CIT0072] Koenig D , Bayer E, Kang J, Kuhlemeier C, Sinha N. 2009. Auxin patterns *Solanum lycopersicum* leaf morphogenesis. Development 136, 2997–3006. 19666826 10.1242/dev.033811

[CIT0073] Kumar R , Kushalappa K, Godt D, Pidkowich MS, Pastorelli S, Hepworth SR, Haughn GW. 2007. The Arabidopsis BEL1-LIKE HOMEODOMAIN proteins SAW1 and SAW2 act redundantly to regulate *KNOX* expression spatially in leaf margins. The Plant Cell 19, 2719–2735. 17873098 10.1105/tpc.106.048769 PMC2048708

[CIT0074] Laufs P , Peaucelle A, Morin H, Traas J. 2004. MicroRNA regulation of the CUC genes is required for boundary size control in *Arabidopsis* meristems. Development 131, 4311–4322. 15294871 10.1242/dev.01320

[CIT0075] Lee JH , Lin H, Joo S, Goodenough U. 2008. Early sexual origins of homeoprotein heterodimerization and evolution of the plant KNOX/BELL family. Cell 133, 829–840. 18510927 10.1016/j.cell.2008.04.028

[CIT0076] Lincoln C , Long J, Yamaguchi J, Serikawa K, Hake S. 1994. A *knotted1*-like homeobox gene in Arabidopsis is expressed in the vegetative meristem and dramatically alters leaf morphology when overexpressed in transgenic plants. The Plant Cell 6, 1859–1876. 7866029 10.1105/tpc.6.12.1859 PMC160567

[CIT0077] Liu Y , You S, Taylor-Teeples M, Li WL, Schuetz M, Brady SM, Douglas CJ. 2014. BEL1-LIKE HOMEODOMAIN6 and KNOTTED ARABIDOPSIS THALIANA7 interact and regulate secondary cell wall formation via repression of *REVOLUTA*. The Plant Cell 26, 4843–4861. 25490916 10.1105/tpc.114.128322 PMC4311193

[CIT0078] Long JA , Moan EI, Medford JI, Barton MK. 1996. A member of the KNOTTED class of homeodomain proteins encoded by the *STM* gene of *Arabidopsis*. Nature 379, 66–69. 8538741 10.1038/379066a0

[CIT0079] Magnani E , Hake S. 2008. *KNOX* lost the *OX*: the Arabidopsis *KNATM* gene defines a novel class of KNOX transcriptional regulators missing the homeodomain. The Plant Cell 20, 875–887. 18398054 10.1105/tpc.108.058495 PMC2390742

[CIT0080] Maksimova AIM , Berke L, Salgado MG, Klimova EA, Pawlowski K, Romanova MA, Voitsekhovskaja OV. 2021. What can the phylogeny of *class I KNOX* genes and their expression patterns in land plants tell us about the evolution of shoot development? Botanical Journal of the Linnean Society 195, 254–280.

[CIT0081] Mallory AC , Dugas DV, Bartel DP, Bartel B. 2004. MicroRNA regulation of NAC-domain targets is required for proper formation and separation of adjacent embryonic, vegetative, and floral organs. Current Biology 14, 1035–1046. 15202996 10.1016/j.cub.2004.06.022

[CIT0082] Molinero-Rosales N , Jamilena M, Zurita S, Gomez P, Capel J, Lozano R. 1999. *FALSIFLORA*, the tomato orthologue of *FLORICAULA* and *LEAFY*, controls flowering time and floral meristem identity. The Plant Journal 20, 685–693. 10652140 10.1046/j.1365-313x.1999.00641.x

[CIT0083] Moyroud E , Kusters E, Monniaux M, Koes R, Parcy F. 2010. LEAFY blossoms. Trends in Plant Science 15, 346–352. 20413341 10.1016/j.tplants.2010.03.007

[CIT0084] Mukherjee K , Brocchieri L, Burglin TR. 2009. A comprehensive classification and evolutionary analysis of plant homeobox genes. Molecular Biology and Evolution 26, 2775–2794. 19734295 10.1093/molbev/msp201 PMC2775110

[CIT0085] Müller J , Wang Y, Franzen R, Santi L, Salamini F, Rohde W. 2001. *In vitro* interactions between barley TALE homeodomain proteins suggest a role for protein–protein associations in the regulation of *Knox* gene function. The Plant Journal 27, 13–23. 11489179 10.1046/j.1365-313x.2001.01064.x

[CIT0086] Nagasaki H , Sakamoto T, Sato Y, Matsuoka M. 2001. Functional analysis of the conserved domains of a rice KNOX homeodomain protein, OSH15. The Plant Cell 13, 2085–2098. 11549765 10.1105/TPC.010113 PMC139453

[CIT0087] Nakayama H , Rowland SD, Cheng Z, Zumstein K, Kang J, Kondo Y, Sinha NR. 2021. Leaf form diversification in an ornamental heirloom tomato results from alterations in two different *HOMEOBOX* genes. Current Biology 31, 4788–4799. 34473947 10.1016/j.cub.2021.08.023

[CIT0088] Nikolov LA , Shushkov P, Nevado B, Gan X, Al-Shehbaz IA, Filatov D, Bailey CD, Tsiantis M. 2019. Resolving the backbone of the Brassicaceae phylogeny for investigating trait diversity. New Phytologist 222, 1638–1651. 30735246 10.1111/nph.15732

[CIT0089] Nikovics K , Blein T, Peaucelle A, Ishida T, Morin H, Aida M, Laufs P. 2006. The balance between the *MIR164A* and *CUC2* genes controls leaf margin serration in *Arabidopsis*. The Plant Cell 18, 2929–2945. 17098808 10.1105/tpc.106.045617 PMC1693934

[CIT0090] Nishimura A , Tamaoki M, Sakamoto T, Matsuoka M. 2000. Over-expression of tobacco *knotted1*-type class1 homeobox genes alters various leaf morphology. Plant and Cell Physiology 41, 583–590. 10929941 10.1093/pcp/41.5.583

[CIT0091] Ori N , Cohen AR, Etzioni A, et al. 2007. Regulation of *LANCEOLATE* by *miR319* is required for compound-leaf development in tomato. Nature Genetics 39, 787–791. 17486095 10.1038/ng2036

[CIT0092] Ori N , Eshed Y, Chuck G, Bowman JL, Hake S. 2000. Mechanisms that control *knox* gene expression in the *Arabidopsis* shoot. Development 127, 5523–5532. 11076771 10.1242/dev.127.24.5523

[CIT0093] Palatnik JF , Allen E, Wu X, Schommer C, Schwab R, Carrington JC, Weigel D. 2003. Control of leaf morphogenesis by microRNAs. Nature 425, 257–263. 12931144 10.1038/nature01958

[CIT0094] Parnis A , Cohen O, Gutfinger T, Hareven D, Zamir D, Lifschitz E. 1997. The dominant developmental mutants of tomato, *Mouse-ear* and *Curl*, are associated with distinct modes of abnormal transcriptional regulation of a *Knotted* gene. The Plant Cell 9, 2143–2158. 9437860 10.1105/tpc.9.12.2143 PMC157064

[CIT0095] Pautot V , Berbel A, Cayla T, Eschstruth A, Adroher B, Ratet P, Madueno F, Laufs P. 2022. *Arabidopsis thaliana SHOOT MERISTEMLESS* substitutes for *Medicago truncatula SINGLE LEAFLET1* to form complex leaves and petals. International Journal of Molecular Sciences 23, 14114. 36430591 10.3390/ijms232214114 PMC9697493

[CIT0096] Piazza P , Bailey CD, Cartolano M, et al. 2010. *Arabidopsis thaliana* leaf form evolved via loss of KNOX expression in leaves in association with a selective sweep. Current Biology 20, 2223–2228. 21129970 10.1016/j.cub.2010.11.037

[CIT0097] Rast-Somssich MI , Broholm S, Jenkins H, et al. 2015. Alternate wiring of a *KNOXI* genetic network underlies differences in leaf development of *A. thalian*a and *C. hirsuta*. Genes and Development 29, 2391–2404. 26588991 10.1101/gad.269050.115 PMC4691893

[CIT0098] Rutjens B , Bao D, van Eck-Stouten E, Brand M, Smeekens S, Proveniers M. 2009. Shoot apical meristem function in Arabidopsis requires the combined activities of three BEL1-like homeodomain proteins. The Plant Journal 58, 641–654. 19175771 10.1111/j.1365-313X.2009.03809.x

[CIT0099] Sakakibara K , Ando S, Yip HK, Tamada Y, Hiwatashi Y, Murata T, Deguchi H, Hasebe M, Bowman JL. 2013. KNOX2 genes regulate the haploid-to-diploid morphological transition in land plants. Science 339, 1067–1070. 23449590 10.1126/science.1230082

[CIT0100] Sakakibara K , Nishiyama T, Deguchi H, Hasebe M. 2008. Class 1 KNOX genes are not involved in shoot development in the moss *Physcomitrella patens* but do function in sporophyte development. Evolution and Development 10, 555–566. 18803774 10.1111/j.1525-142X.2008.00271.x

[CIT0101] Sakamoto T , Nishimura A, Tamaoki M, Kuba M, Tanaka H, Iwahori S, Matsuoka M. 1999. The conserved KNOX domain mediates specificity of tobacco KNOTTED1-type homeodomain proteins. The Plant Cell 11, 1419–1432. 10449577 10.1105/tpc.11.8.1419 PMC144289

[CIT0102] Satterlee JW , Scanlon MJ. 2019. Coordination of leaf development across developmental axes. Plants 8, 433. 31652517 10.3390/plants8100433 PMC6843618

[CIT0103] Scarpella E , Marcos D, Friml J, Berleth T. 2006. Control of leaf vascular patterning by polar auxin transport. Genes and Development 20, 1015–1027. 16618807 10.1101/gad.1402406 PMC1472298

[CIT0104] Scofield S , Murison A, Jones A, Fozard J, Aida M, Band LR, Bennett M, Murray JAH. 2018. Coordination of meristem and boundary functions by transcription factors in the SHOOT MERISTEMLESS regulatory network. Development 145, dev157081. 29650590 10.1242/dev.157081 PMC5992597

[CIT0105] Semiarti E , Ueno Y, Tsukaya H, Iwakawa H, Machida C, Machida Y. 2001. The *ASYMMETRIC LEAVES2* gene of *Arabidopsis thaliana* regulates formation of a symmetric lamina, establishment of venation and repression of meristem-related homeobox genes in leaves. Development 128, 1771–1783. 11311158 10.1242/dev.128.10.1771

[CIT0106] Serikawa KA , Zambryski PC. 1997. Domain exchanges between KNAT3 and KNAT1 suggest specificity of the kn1-like homeodomains requires sequences outside of the third helix and N-terminal arm of the homeodomain. The Plant Journal 11, 863–869. 9161041 10.1046/j.1365-313x.1997.11040863.x

[CIT0107] Serra L , Perrot-Rechenmann C. 2020. Spatio temporal control of cell growth by CUC3 shapes leaf margins. Development 147, dev183277. 32094116 10.1242/dev.183277

[CIT0108] Shani E , Burko Y, Ben-Yaakov L, Berger Y, Amsellem Z, Goldshmidt A, Sharon E, Ori N. 2009. Stage-specific regulation of *Solanum lycopersicum* leaf maturation by class 1 KNOTTED1-LIKE HOMEOBOX proteins. The Plant Cell 21, 3078–3092. 19820191 10.1105/tpc.109.068148 PMC2782295

[CIT0109] Sicard A , Thamm A, Marona C, Lee YW, Wahl V, Stinchcombe JR, Wright SI, Kappel C, Lenhard M. 2014. Repeated evolutionary changes of leaf morphology caused by mutations to a homeobox gene. Current Biology 24, 1880–1886. 25127212 10.1016/j.cub.2014.06.061

[CIT0110] Sieber P , Wellmer F, Gheyselinck J, Riechmann JL, Meyerowitz EM. 2007. Redundancy and specialization among plant microRNAs: role of the *MIR164* family in developmental robustness. Development 134, 1051–1060. 17287247 10.1242/dev.02817

[CIT0111] Sinha NR , Williams RE, Hake S. 1993. Overexpression of the maize homeo box gene, *KNOTTED-1*, causes a switch from determinate to indeterminate cell fates. Genes and Development 7, 787–795. 7684007 10.1101/gad.7.5.787

[CIT0112] Smith HM , Boschke I, Hake S. 2002. Selective interaction of plant homeodomain proteins mediates high DNA-binding affinity. Proceedings of the National Academy of Sciences, USA 99, 9579–9584. 10.1073/pnas.092271599 PMC123183 12093897

[CIT0113] Smith HM , Hake S. 2003. The interaction of two homeobox genes, *BREVIPEDICELLUS* and *PENNYWISE*, regulates internode patterning in the Arabidopsis inflorescence. The Plant Cell 15, 1717–1727. 12897247 10.1105/tpc.012856 PMC167164

[CIT0114] Smith LG , Greene B, Veit B, Hake S. 1992. A dominant mutation in the maize homeobox gene, *Knotted-1*, causes its ectopic expression in leaf cells with altered fates. Development 116, 21–30. 1362381 10.1242/dev.116.1.21

[CIT0115] Sousa-Baena MS , Lohmann LG, Rossi M, Sinha NR. 2014. Acquisition and diversification of tendrilled leaves in Bignonieae (Bignoniaceae) involved changes in expression patterns of *SHOOTMERISTEMLESS (STM), LEAFY/FLORICAULA* (*LFY/FLO*), and *PHANTASTICA* (*PHAN*). New Phytologist 201, 993–1008. 24237175 10.1111/nph.12582

[CIT0116] Spinelli SV , Martin AP, Viola IL, Gonzalez DH, Palatnik JF. 2011. A mechanistic link between *STM* and *CUC1* during Arabidopsis development. Plant Physiology 156, 1894–1904. 21685178 10.1104/pp.111.177709 PMC3149926

[CIT0117] Tamaoki M , Kusaba S, Kano-Murakami Y, Matsuoka M. 1997. Ectopic expression of a tobacco homeobox gene, *NTH15*, dramatically alters leaf morphology and hormone levels in transgenic tobacco. Plant and Cell Physiology 38, 917–927. 9327591 10.1093/oxfordjournals.pcp.a029252

[CIT0118] Tattersall AD , Turner L, Knox MR, Ambrose MJ, Ellis TH, Hofer JM. 2005. The mutant *crispa* reveals multiple roles for *PHANTASTICA* in pea compound leaf development. The Plant Cell 17, 1046–1060. 15749758 10.1105/tpc.104.029447 PMC1087985

[CIT0119] Vlad D , Kierzkowski D, Rast MI, et al. 2014. Leaf shape evolution through duplication, regulatory diversification, and loss of a homeobox gene. Science 343, 780–783. 24531971 10.1126/science.1248384

[CIT0120] Vollbrecht E , Veit B, Sinha N, Hake S. 1991. The developmental gene *Knotted-1* is a member of a maize homeobox gene family. Nature 350, 241–243. 1672445 10.1038/350241a0

[CIT0121] Vroemen CW , Mordhorst AP, Albrecht C, Kwaaitaal MA, de Vries SC. 2003. The *CUP-SHAPED COTYLEDON3* gene is required for boundary and shoot meristem formation in Arabidopsis. The Plant Cell 15, 1563–1577. 12837947 10.1105/tpc.012203 PMC165401

[CIT0122] Vuolo F , Mentink RA, Hajheidari M, Bailey CD, Filatov DA, Tsiantis M. 2016. Coupled enhancer and coding sequence evolution of a homeobox gene shaped leaf diversity. Genes and Development 30, 2370–2375. 27852629 10.1101/gad.290684.116 PMC5131777

[CIT0123] Wang H , Chen J, Wen J, Tadege M, Li G, Liu Y, Mysore KS, Ratet P, Chen R. 2008. Control of compound leaf development by *FLORICAULA/LEAFY* ortholog *SINGLE LEAFLET1* in *Medicago truncatula*. Plant Physiology 146, 1759–1772. 18287485 10.1104/pp.108.117044 PMC2287348

[CIT0124] Wang Y , Sören S, Shanda L, Bjorn P, Rena L, Adam R, Miltos T. 2022. The cellular basis for synergy between *RCO* and *KNOX1* homeobox genes in leaf shape diversity. Current Biology 32, 3773–3784. 36029772 10.1016/j.cub.2022.08.020

[CIT0125] Xiong Y , Jiao Y. 2019. The diverse roles of auxin in regulating leaf development. Plants 8, 243. 31340506 10.3390/plants8070243 PMC6681310

[CIT0126] Xu L , Xu Y, Dong A, Sun Y, Pi L, Xu Y, Huang H. 2003. Novel *as1* and *as2* defects in leaf adaxial–abaxial polarity reveal the requirement for *ASYMMETRIC LEAVES1* and *2* and *ERECTA* functions in specifying leaf adaxial identity. Development 130, 4097–4107. 12874130 10.1242/dev.00622

[CIT0127] Yu C , Yan C, Liu Y, et al. 2020. Upregulation of a *KN1* homolog by transposon insertion promotes leafy head development in lettuce. Proceedings of the National Academy of Sciences, USA 117, 33668–33678. 10.1073/pnas.2019698117 PMC7776633 33288708

[CIT0128] Yu H , Zhang L, Wang W, et al. 2020. TCP5 controls leaf margin development by regulating the KNOX and BEL-like transcription factors in Arabidopsis. Journal of Experimental Botany 72, 1809–1821. 10.1093/jxb/eraa569 33258902

[CIT0129] Zhou C , Han L, Hou C, Metelli A, Qi L, Tadege M, Mysore KS, Wang ZY. 2011. Developmental analysis of a *Medicago truncatula smooth leaf margin1* mutant reveals context-dependent effects on compound leaf development. The Plant Cell 23, 2106–2124. 21693694 10.1105/tpc.111.085464 PMC3160044

[CIT0130] Zhou C , Han L, Li G, Chai M, Fu C, Cheng X, Wen J, Tang Y, Wang ZY. 2014. STM/BP-Like KNOXI is uncoupled from ARP in the regulation of compound leaf development in *Medicago truncatula*. The Plant Cell 26, 1464–1479. 24781113 10.1105/tpc.114.123885 PMC4036565

[CIT0131] Zhu QH , Zhang J, Liu D, Stiller W, Liu D, Zhang Z, Llewellyn D, Wilson I. 2016. Integrated mapping and characterization of the gene underlying the okra leaf trait in *Gossypium hirsutum* L. Journal of Experimental Botany 67, 763–774. 26567355 10.1093/jxb/erv494 PMC4737076

